# Data on common carotid artery occlusion inducing focalized stroke lesions after Pertussis toxin injection

**DOI:** 10.1016/j.dib.2022.108851

**Published:** 2022-12-25

**Authors:** Veronika Leiss, Roland P. Piekorz, Salvador Castaneda Vega, Christoph Jacoby, Ulrich Flögel, Katja Pexa, Jürgen Schrader, Bernd J. Pichler, Sandra Beer-Hammer, Bernd Nürnberg

**Affiliations:** a Department of Pharmacology, Experimental Therapy and Toxicology, and Interfaculty Center for Pharmacogenomics and Drug Research, Eberhard Karls University Tübingen and University Clinic, Tübingen, Germany; b Institute of Biochemistry and Molecular Biology II, Medical Faculty, Heinrich Heine University Düsseldorf, Düsseldorf, Germany; c Werner Siemens Imaging Center, Department of Preclinical Imaging and Radiopharmacy, Eberhard Karls University Tübingen and University Clinic, Tübingen, Germany; d Department of Nuclear Medicine and Clinical Molecular Imaging, Eberhard Karls University Tübingen and University Clinic, Tübingen, Germany; e Institute of Molecular Cardiology, Medical Faculty, Heinrich Heine University Düsseldorf, Düsseldorf, Germany; f Cluster of Excellence iFIT (EXC 2180) “Image-Guided and Functionally Instructed Tumor Therapies”, Eberhard Karls University, Tübingen, Germany

**Keywords:** Angiography, G_i_ proteins, ischemic stroke, murine model, PTX, Pertussis Toxin, CCAO, Common carotid artery occlusion

## Abstract

This article contains raw and processed data related to research published by Vega et al. (2022). This complementary dataset provides further insight into the experimental validation of a single common carotid artery occlusion (CCAO) model upon pretreatment with pertussis toxin (PTX). We present data showing the extent of different PTX concentrations on neurological severity measured by Bederson score following CCAO. In addition, data indicate a protective effect of isoflurane on cerebral infarction and neurological deficits, as well as the consequences of PTX pretreatment on reperfusion after occlusion using time-of-flight magnetic resonance angiography. With these data, we aim to provide detailed experimental settings of this newly described model.

**Specifications Table**

**Table:** 

Subject	Health and medical sciences
Specific subject area	Cardiology and Cardiovascular Medicine
Type of data	Table, Figure, Video
How the data were acquired	Vertical Bruker DRX 9.4 T wide-bore NMR spectrometer, a 40-mm gradient set (capable of 1 T/m maximum gradient strength) and a linearly driven 30-mm birdcage resonator
Data format	Raw, analyzed
Description of data collection	PTX pretreated C57BL/6N mice were purchased from Jackson Laboratories and used as experimental animals. Time-of-flight angiography was conducted with the vertical Bruker DRX 9.4 T wide-bore NMR spectrometer (Bruker, Billerica; MA, USA). Neurological deficits were assessed with a modified Bederson score.
Data source location	• Institution: Institute of Biochemistry and Molecular Biology II, Medical Faculty, Heinrich Heine University Düsseldorf • City/Town/Region: Düsseldorf • Country: Germany • Institution: Department of Pharmacology, Experimental Therapy and Toxicology, and Interfaculty Center for Pharmacogenomics and Drug Research, Eberhard Karls University Tübingen and University Clinic • City/Town/Region: Tübingen • Country: Germany
Data accessibility	Data are with this article; http://dx.doi.org/10.17632/46ddhz7g9d.2 https://data.mendeley.com/datasets/46ddhz7g9d/2
Related research article	S. Castaneda‑Vega, S. Beer‑Hammer, V. Leiss, H. Napieczyńska, M. Vuozzo, A.M. Schmid, H. Zeng, Y. He, U. Kohlhofer, I. Gonzalez‑Menendez, L. Quintanilla‑Martinez, J.M. Hempel, M. Gollasch, X. Yu, B.J. Pichler, B. Nürnberg, Cerebrovascular Gi Proteins Protect Against Brain Hypoperfusion and Collateral Failure in Cerebral Ischemia. Molecular Imaging and Biology https://doi.org/10.1007/s11307-022-01764-8

**Value of the Data**

• Dataset is thought to be used for the evaluation of the novel ischemic stroke model described in Vega *et al.* (2022) [1].
• Data contains further experimental validation of common carotid artery occlusion (CCAO) under various PTX conditions (concentrations and incubation times).
• Data will be useful to researchers who use PTX to inhibit intracellular signaling *via* heterotrimeric G_i_ and G_o_ proteins, especially in the context of neuronal signaling.
• Data could assist researchers in choosing the appropriate anesthetic drug.
• Data also indicate the protective effect of isoflurane during reperfusion on neurological outcome following CCAO in PTX-treated mice.

## 1 Objective

This dataset was generated to compare different doses of PTX and anesthetics regarding the outcome of CCAO. It will serve as “material and methods” for several future articles that will investigate CCAO using PTX as a tool to perturb G_i_- and G_o_-mediated signaling pathways. Ultimately, the objective is to determine the most suitable combination of PTX and anesthetics in mice undergoing CCAO to replace, reduce and refine in the sense of the 3R principle. The data indicate the administration of 90 µg PTX per kilogram body weight 48 h before the intervention as the optimal condition to induce cerebral stroke.

## 2 Data Description

**Fig. 1:**
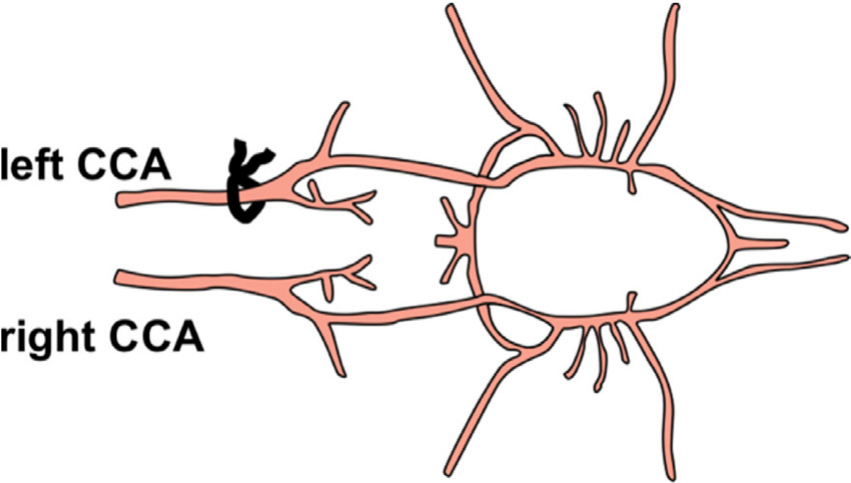
Scheme of left common carotid artery occlusion. Schematic representation of the major mouse head (right) and neck (left) arteries (view from the top) and the appropriate position of the non-absorbable suture (black loop) during left common carotid artery (CCA) occlusion.

**Fig. 2:**
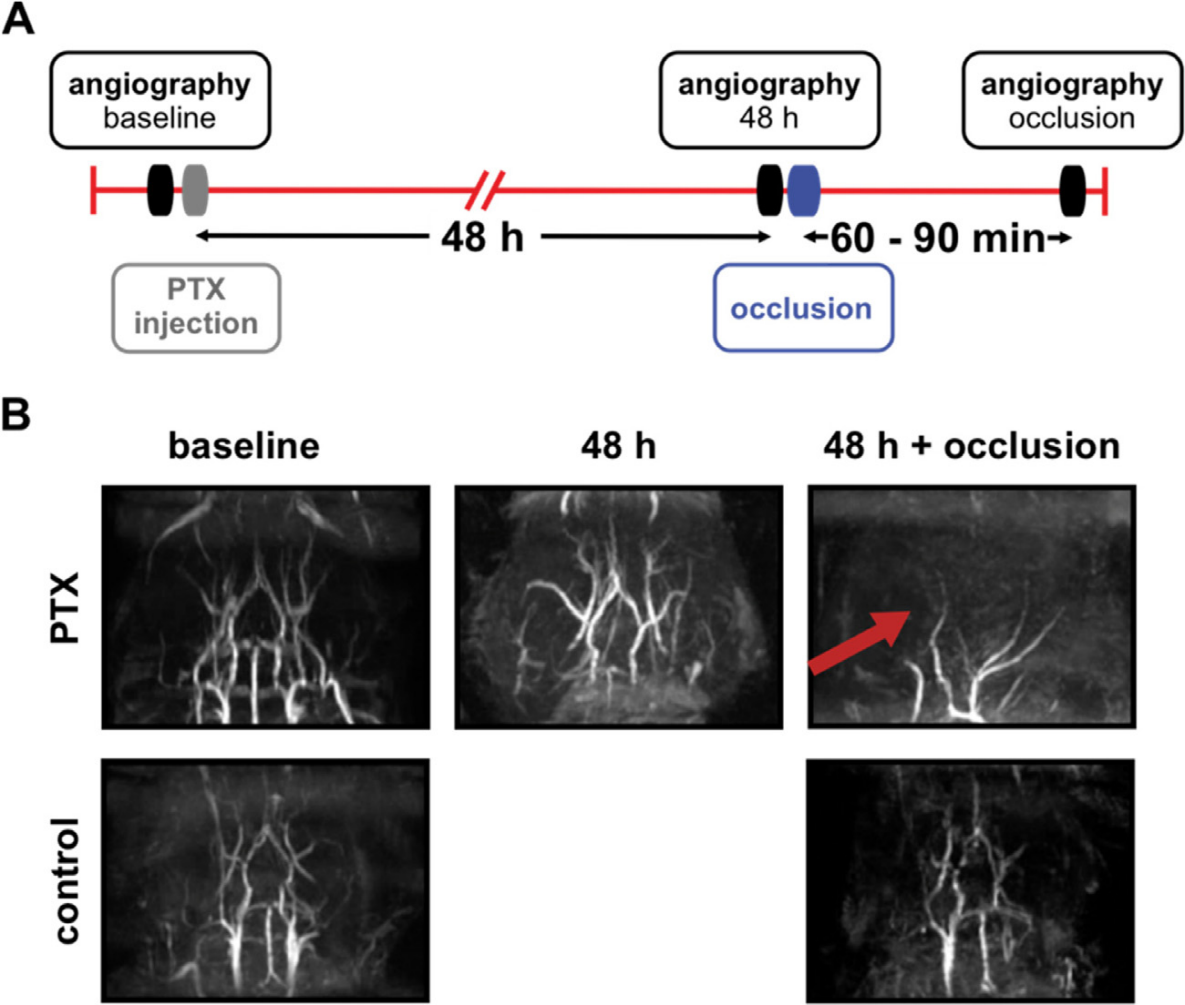
Time-of-flight magnetic resonance angiography in mice following i.p. injection of PTX and subsequent CCA occlusion. A. Scheme showing time points of MRI analysis, PTX injection (90 µg/kg b.w. i.p.) and CCA occlusion. B. Isoflurane-anesthetized mice (n = 5) underwent angiography of cerebral blood flow (baseline, left panel). Subsequently, four mice received a PTX injection. In these mice a second MRI-image of cerebral blood flow was measured 48 h later (48 h, middle panel). Immediately afterwards, the left CCA was permanently ligated, and the mice were subjected to a third round of MRI (48 h + occlusion, upper panel, right). As a control non-treated mice were scanned again after CCAO (lower panel, right). Shown are images of one representative mouse of each group. The red arrow indicates the disrupted blood flow.

**Fig. 3:**
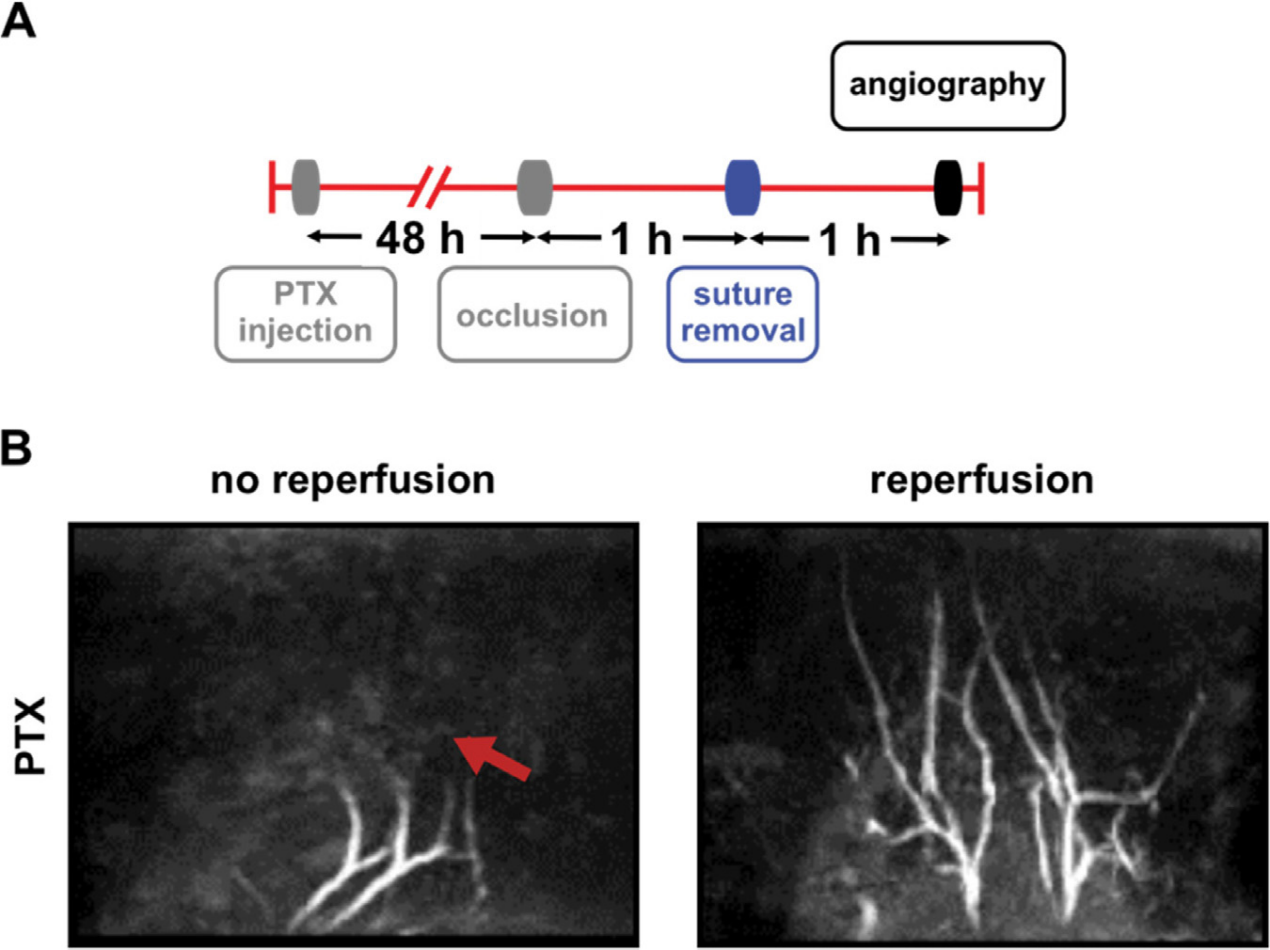
TOF MRI in mice following i.p. injection of PTX, CCA ligation, and subsequent removal of the non-absorbable suture. A. Scheme showing time points of PTX injection (90 µg/kg b.w. i.p.), CCA ligation for one hour (occlusion), suture removal, one hour reperfusion, and angiography. B. Isoflurane-anesthetized mice (n = 8) underwent angiography one hour after the removal of the suture. Shown are representative images of a mouse showing no reperfusion (left image, red arrow) and a mouse demonstrating successful reperfusion (right image).

In this work we investigated the consequences of PTX pretreatment on cerebral blood flow by time-of-flight (TOF) angiography before, during and after unilateral common carotid artery occlusion (CCAO) (Figs.1-3, Table 1, Videos 1-5). In addition, different doses of PTX with different incubation times were used (Table 2). Table 3 shows the effect of isoflurane duration whereas Table 4 depicts the effect of isoflurane- or fentanyl-based anesthesia on the severity score of neurological deficits.

**Table 1:** Modified Bederson score.

Neurological severity level	Neurological deficits
0	no deficits
1	flexion of front paw
2	flexion of front paw and impaired lateral positioning reflex
3	circling
4	circling and spinning
5	immobilisation

**Table 2:** Effect of various PTX treatments on neurological deficits in mice with unilateral CCA occlusion. Animals received indicated PTX doses (A - 48–72 h; B - 96–120 h) or B-oligomer of PTX (C - 48–72 h) prior to occlusion or sham surgery. Neurological deficits were assessed immediately after the mice woke up from isoflurane anesthesia. Isoflurane treatment was less than 15 min. Numbers and percentages of mice showing neurological deficits are depicted. PTX-pretreated sham-operated mice showed no neurological deficits.

PTX (µg/kg b.w.)		Occlusion (frequency)	Sham (frequency)
A	50 – 75	0/4 (0%)	0/2 (0%)
	90 – 100	18/20 (90%)	0/11 (0%)
	150	5/5 (100%)	0/1 (0%)
B	30 – 75	1/4 (25%)	0/2 (0%)
	150 – 300	6/6 (100%)	0/2 (0%)
C	150	0/3 (0%)	0/3 (0%)

**Table 3:** Effect of the duration of isoflurane-based anesthesia on the severity of neurological deficits in PTX-pretreated, transiently ligated mice. Mice were injected with PTX (90 µg/kg b.w. i.p.) 48 h prior to a transient ligation of the left carotid artery under isoflurane anesthesia for the indicated time periods. Thereafter, ligation was removed, and one group of mice was additionally anesthetized with isoflurane for another 30 min (grey shading). Thereafter all mice underwent Bederson scoring (“severity score”) for assessment of neurological deficits. Prolongation of isoflurane anesthesia reduced the severity score.

PTX (90 µg/kg b.w.)	Duration of transient ligation under 2% isoflurane	2% isoflurane during reperfusion	Severity score
+	30 min	-	4
+	15 min	-	3
+	15 min	-	4
+	15 min	-	3
+	30 min	30 min	1
+	30 min	30 min	2
+	30 min	30 min	1
+	30 min	30 min	2

**Table 4:** Effect of the duration of isoflurane- or fentanyl-based anesthesia on the severity of neurological deficits in PTX-pretreated occluded mice. Mice were injected with PTX (90 µg/kg b.w. i.p.) 48 h prior to a CCAO under isoflurane (grey shading) or fentanyl-based anesthesia for 30 min. Thereafter all mice underwent modified Bederson scoring (“severity score”) for assessment of neurological deficits. Isoflurane anesthesia reduced the severity score.

PTX (90 µg/kg b.w.)	Duration of transient ligation		Severity score
	2% isoflurane	fentanyl-based	
+	30 min		0
+	30 min		0
+	30 min		0
+	30 min		3
+	30 min		0
+	30 min		2
+	30 min		2
+	30 min		3
+	30 min		0
+	30 min		0
+		30 min	5
+		30 min	2
+		30 min	3
+		30 min	4
+		30 min	3
+		30 min	2
+		30 min	3
+		30 min	0
+		30 min	5

## 3 Experimental Design, Materials and Methods

### 3.1 Animals

The study was carried out in compliance with the ARRIVE guidelines. All experiments were performed according to the EU Animals Scientific Procedures Act and the German law for the welfare of animals and were approved by the local animal ethical committee Bezirksregierung Düsseldorf (8.87-50.10.34.08.018) and Bezirksregierung Tübingen (PH2/17). C57BL/6 female mice were kept under specified pathogen-free conditions, controlled temperature and humidity in 12-h day/night light cycle and received food and water ad libitum.

### 3.2 Anesthesia

For surgeries and imaging experiments, animals were anaesthetized using isoflurane (induction at 2.5% and maintenance at 2% vaporized in 100% O_2_ at a flow rate of 1.5 L/min) and placed on an MRI-compatible bed with an integrated water heating system (Bruker Biospin). Fentanyl 0.05 mg/kg body weight (b.w.), midazolam 5.0 mg/kg b.w., medetomidine 0.5 mg/kg b.w. narcosis was injected intraperitoneally and used on anesthesia-focused experiments as indicated.

### 3.3 Common carotid artery occlusion

The animals were dissected under a surgical microscope through a small median incision above the trachea through the submaxillary salivary glands, exposing the left common carotid artery (CCA). The CCA was carefully dissected bypassing the Vagus nerve. Transient occlusion of the CCA was achieved using a non-absorbable suture (Ethicon, Norderstedt-Glashütte, Germany) (Fig. 1). Considering the opening and closing of the surgical wound, each procedure took about 10 to 15 minutes. The mice were allowed to recover in their home cages before they were assessed by a modified Bederson score (Table 1) and scanned 60 to 90 min after the procedure.

### 3.4 Bederson score

Neurological deficits were determined using a modified Bederson score as depicted in Table 1 [2,3].

### 3.5 Time-of-flight magnet resonance angiography

Time-of-flight (TOF) angiography is an MRI technique to visualize flow within vessels without administering contrast agents. Mice were examined between 8 to 14 weeks of age. Angiographies were carried out under isoflurane anaesthesia using an established TOF imaging protocol and a vertical Bruker DRX 9.4 T wide-bore NMR spectrometer, a 40-mm gradient set (capable of 1 T/m maximum gradient strength) and a linearly driven 30-mm birdcage resonator [4].

Within a first setup (Fig. 2) baseline angiography was performed (Videos 1,2), mice were subsequently injected i.p. with PTX (90 µg/kg b.w. (previously shown in [5]); Merck KGaA, Darmstadt, Germany), and scanned after 48 h (Video 3). A permanent ligation of the left carotid artery was performed, and animals were allowed to recover for 60 to 90 min, assessed by Bederson score, and scanned again (Video 4,5).

Within a second set of experiments mice were pretreated with PTX and the CCA was ligated (Fig. 3A). In addition, neurological deficits were Bederson scored demonstrating that all mice displayed a stroke phenotype (Bederson score ≥ 1). Subsequently, ligation was removed and after one hour of reperfusion mice underwent MRI analysis to evaluate cerebral blood flow. While all mice showed a stroke phenotype, only three out of six mice demonstrated reperfusion (Fig. 3B).

## Ethics Statements

The study was carried out in compliance with the ARRIVE guidelines. All experiments were performed according to the EU Directive 2010/63/EU and the German law for the welfare of animals and were approved by the local animal ethical committee Bezirksregierung Düsseldorf und Tübingen.

## CRediT authorship contribution statement

**Veronika Leiss:** Investigation, Visualization, Writing – original draft, Writing – review & editing. **Roland P. Piekorz:** Investigation, Writing – original draft, Writing – review & editing. **Salvador Castaneda Vega:** Investigation, Writing – original draft. **Christoph Jacoby:** Methodology, Software, Investigation. **Ulrich Flögel:** Methodology, Software. **Katja Pexa:** Investigation. **Jürgen Schrader:** Methodology, Software. **Bernd J. Pichler:** Supervision, Writing – original draft. **Sandra Beer-Hammer:** Investigation, Visualization, Writing – original draft, Writing – review & editing. **Bernd Nürnberg:** Conceptualization, Supervision, Writing – review & editing.

## Declaration of Competing Interest

The authors declare that they have no known competing financial interests or personal relationships that could have appeared to influence the work reported in this paper.

## Data Availability

Dataset of supporting videos to PTX-stroke study (Original data) (Mendeley Data).

## Appendix Supplementary materials

**Video 1. 3D-Angiography of a wild-type mouse before PTX-injection.** Video analysis of MRI angiography is presented for the representative mouse analyzed and depicted in Fig. 2B (upper panel, left). Image, video 1

**Video 2. 3D-Angiography of a wild-type mouse without PTX-injection**. Video analysis of MRI angiography is presented for the representative mouse analyzed and depicted in Fig. 2B (lower panel, left). Image, video 2

**Video 3. 3D-Angiography of a wild-type mouse 48 h after PTX-injection.** Video analysis of MRI angiography is presented for the representative mouse analyzed and depicted in Fig. 2B (upper panel, middle). Image, video 3

**Video 4. 3D-Angiography of a wild-type mouse 48 h after PTX-injection and subsequent CCAO.** Video analysis of MRI angiography is presented for the representative mouse analyzed and depicted in Fig. 2B (upper panel, right). Image, video 4

**Video 5. 3D-Angiography of a wild-type mouse without PTX-injection but subjected to CCAO.** Video analysis of MRI angiography is presented for the representative mouse analyzed and depicted in Fig. 2B (lower panel, right). Image, video 5
